# Prioritizing evidence for action from the 2024 small island developing states report of the *Lancet* Countdown on health and climate change

**DOI:** 10.1016/j.joclim.2025.100482

**Published:** 2025-07-23

**Authors:** Stephanie Y Parker, Kimalie F Parchment, Maria Walawender, Georgiana Gordon-Strachan

**Affiliations:** a Caribbean Institute for Health Research, Tropical Metabolism Research Unit, University of the West Indies, Kingston, Jamaica; b Institute for Global Health, *Lancet* Countdown, University College of London, London W1T 4TJ, UK

**Keywords:** Small island developing states, Climate change and health, Climate impacts, Health systems, Adaptation

## Abstract

**Introduction:**

Small island developing states (SIDS) are vulnerable to the ill-health effects associated with unabated atmospheric warming driven by larger, more developed countries. Hence, the health-centered focus of the *Lancet* Countdown on Health and Climate Change has immense value for SIDS.

**Methods:**

Given resource constraints and data limitations across the SIDS region, the inaugural 2024 SIDS report of the *Lancet* Countdown required a method of singling out the most regionally relevant indicators from 47 *Lancet* Countdown global indicators. A two-stage semi-qualitative method was designed for this purpose.

**Results:**

The evaluation stage involved identifying two indicator sets from stakeholder consultation (*n* = 32) and a multi-criteria assessment (*n* = 11). Using both sets of indicators, only 11 met the criteria of being regionally representative and relevant, further confirming data limitations. To adjust for this challenge, 14 indicators were added to the final list based on relevance. Therefore, 25 *Lancet* Countdown indicators were selected for the 2024 SIDS report of the *Lancet* Countdown on Health and Climate Change. To further prioritize the wide range of evidence from these indicators, a qualitative multiplication matrix was used to define relationships between regional data coverage and differences from global benchmarks. Six indicators marked successes in adaptation, and 14 evidenced negative implications compared to global benchmarks.

**Conclusion:**

The impact of heat on physical activity, food security, and economic development was of critical regional importance, as was the need for more research. The study identified critical areas where urgent climate action is needed, emphasizing the need for increased research and data collection in SIDS.

## Highlights

• Data & resource constraints can hinder climate resilience in small islands.
• Climate & health indicator evidence can be used for targeted climate action.
• The method in this study ranks evidence by importance for regional decision-making.
• Urgent action is needed for heat impact on SIDS’ productivity & food security.
• Investment is needed to increase health and climate research on SIDS.

## 1 Introduction

Climate change is specifically concerning given the role of human activity in accelerating global climate change and the fact that the associated effects have not been within the collective human experience [1]. These effects embody human vulnerability, exerted through food and water insecurity [2], the spread of disease [3], the exacerbation of pre-existing non-communicable diseases [4], and poor mental health [[4], [5], [6]]. The global scale of this crisis has justified the statement that ‘climate change is the biggest health threat of the 21^st^ century’ [7]. The international community has benefited from a global-scale acceleration of climate change awareness. The 2030 Agenda for Sustainable Development and the Paris Agreement [8], represent the most recent global consensus on the social, economic, and environmental value of limiting atmospheric temperature to 2 °C below pre-industrial levels by 2100.

Climate change amplifies health and development inequities more acutely in less developed and small island states [9]. The smaller economies and populations, limited natural resources, and external dependence of small island states are precursors of climate vulnerability [5,10]. Climate change exerts unavoidable pressure on water and food availability, energy access, and health systems in small island developing states (SIDS) [1,5,11]. Extreme weather events typical to SIDS, such as droughts, storms, or flooding, have displayed changes in duration, intensity, and periodicity that affect well-being and have resulted in death [12,13]. Cessation of greenhouse gas emissions, driven by larger, more developed countries [14], is lagging at the expense of health in SIDS. However, SIDS spearhead the call for climate action internationally [13].

**Fig. 1:**
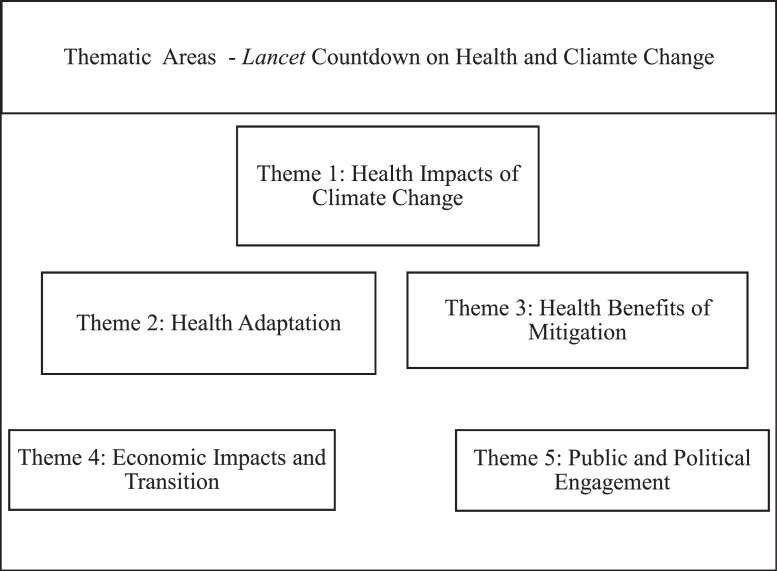
Five thematic areas of the Lancet Countdown on Health and Climate Change Indicator Framework.

The *Lancet* Countdown on Health and Climate Change emerged as an academic movement to track progress towards the 2015 Paris Agreement from the viewpoint of the implications, beneficial or deleterious, on human health [7]. The tenets of the Countdown are to determine the major threats, direct or indirect, to global health from climate change [15]. Furthermore, while mitigation was established as the primary avenue for decarbonizing, other reports acknowledged that decarbonization is as much, or even more so, a political discourse as an economic or technical one. Through examining this consensus on climate change, the five pillars of the Lancet Countdown on Health and Climate Change provided the basis for their indicator framework (Fig. 1) [13].

As of 2024, their indicator framework consisted of 51 indicators across the five thematic areas (Fig. 1;), which address core health concerns of climate change and the health co-benefits of climate action [13]. Recognizing the need for regional perspectives, the *Lancet* Countdown on Health and Climate Change Regional Centre for Small Island Developing States was established in 2022 as one of six centers. Fifty-nine UN member states and associate member states (see supplementary material Table 1) are under the purview of the SIDS Regional Centre based at the Caribbean Institute for Health Research in Jamaica.

The *Lancet* Countdown indicator framework is invaluable for aggregating measurable high-level climate targets. However, global aggregation tends to modulate signals from larger countries and drown out those of smaller states. Data challenges in developing countries and small island states limit the use of global indicator frameworks for strong evidence-based decision-making.

Quantitative methods provide definitive evidence for decision-making and have been successfully applied to policy making, such as health care standards or socio-demographic interventions. As evidence collection becomes more complex, results from quantitative methods like statistical analysis, regression analysis, time-series forecasting, or cost-benefit analysis have been more widely favored for facilitating big data processing in systematic decision-making [16]. Moreover, improvements in health research have driven this nudge towards using numerical methods to condense swaths of data [17]. Quantitative methods may not always be suitable for regional policy and decision-making due to limitations such as no data, low data quality (resolution or inaccuracies), or infrequent data reporting [17] which are common in SIDS.

In contrast, qualitative methods, another subset of empirical research, consist of tools such as participant/expert observation [18]. Qualitative methods can help determine why promising public interventions may or may not have their desired effect [18,19] and can help give voice to overlooked, smaller, or marginalized groups that would have their perceptions obscured by generalizations skewed towards larger groups [20]. However, some scholars point out that qualitative methods are better for smaller-scale assessments and may not be appropriate as a standalone approach depending on the number of parameters being dealt with [16,18]. In these instances, semi-qualitative methods may better facilitate decision-making through the incorporation of publicly available data bolstered by stakeholder expertise or other non-numeric data [16]. In hybridizing, expert opinion can be supported by numerical evidence [21]. The evaluation and ranking method used in this study was developed to identify the most relevant indicators from the *Lancet* Countdown Indicator Framework for use in policymaking by SIDS and was designed for and applied to the 2024 SIDS report [22]. The method aimed to evaluate available evidence based on relevance to the region and to determine the most critical areas needing immediate climate action.

## 2 Methodology

The two-stage evaluation and prioritization methodology was designed to identify the most relevant indicators at a regional scale. The *Lancet* Countdown on Health and Climate Change Indicator Framework was the exemplar for this methodology and validated the evaluation and prioritization stages.

### 2.1 Evaluation stage

**Fig. 2:**
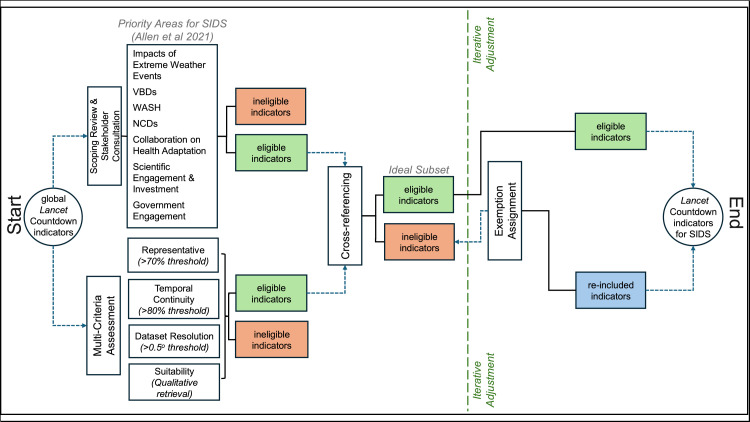
Schematic of the semi-qualitative evaluation method applied to the *Lancet* Countdown Indicator Framework for the SIDS region. This graphic shows the process by which indicators were selected for the 2024 small island developing states report of the *Lancet* Countdown from the larger global *Lancet* Countdown indicator framework.

Four main steps were undertaken in the evaluation stage (schematic depicted in Fig. 2): i) scoping review and stakeholder consultation, ii) multi-criteria assessment, iii) cross-referencing, and iv) exemption assignment. Stakeholder consultation and the multi-criteria assessment were two independent processes. For cross-referencing, overlapping indicators from both processes were carried forward. Finally, exemptions were assigned as too few indicators were obtained.

#### 2.1.1 Scoping review and stakeholder consultation

A scoping review and stakeholder consultation were performed as two parts of a single process aimed at placing expert knowledge from SIDS’ representatives within the global climate and health context. In this case, the literature reviewed was the compendium of knowledge compiled by the UCL-*Lancet* Commission to formulate the five thematic areas used as the basis of the *Lancet* Countdown indicator framework [15]. The review used in this study included a scoping review of the literature as well as a stakeholder consultation of SIDS representatives from the fields of climate change and health, designed to distil the climate consensus specific to SIDS and orient the health elements gleaned from the consultation with the existing *Lancet* Countdown Indicator Framework.

Certain conditions in stakeholder consultations are required to retrieve well-ordered, relevant information. The *Lancet* Countdown on Health and Climate Change, through the host university – UCL, enlisted Blue Sky Development Consulting, a specialist Caribbean agency dedicated to developing evidence-based solutions to public health and social challenges in SIDS, to undertake the stakeholder consultation [23]. Objectives were to identify climate change and health priorities for SIDS and determine the relevance of existing *Lancet* Countdown indicators. After reviewing scientific articles on the subject matter, these stakeholders were purposively contacted through introductory emails from UCL with information on the objective of the consultation. Snowball sampling was then conducted following recommendations from the email respondents. Researchers from Blue Sky, aided by UCL and the Caribbean Institute for Health Research (CAIHR), conducted virtual interviews with 24 key SIDS stakeholders – 17 Caribbean and 7 Pacific representatives. Interviewees consisted of 10 women and 14 men from universities, regional agencies, a UN agency, civil society organizations, regional health agencies, regional climate agencies, Ministries of Health, and a climate research institution. The interviews were organized in two-person research teams where one researcher conducted the interview and the other recorded responses. Semi-structured and open-ended questionnaires were developed to facilitate guided analysis, with the Lancet Countdown thematic areas providing the basis for content analysis. Two other objectives of the stakeholder consultation were to identify areas of relevance to SIDS lacking in the existing indicator framework, as well as relevant capacity gaps in SIDS. Respondents consistently identified data challenges, which were reported for future indicator development. Eight priority areas and associated indicators of relevance to the region were identified in the existing framework based on the interviewees’ responses [23] (see supplementary material Table 2).

#### 2.1.2 Multi-criteria assessment

All 47 indicators in the *Lancet* Countdown Indicator Framework were assessed with the aim of retrieving indicators capable of providing a health and climate situational report for the entire SIDS region. This step is critical for determining the decision-making capacity of available data since results from composite indicators are heavily dependent on the quality of the input data [24]. Studies have shown that the quality of indicators is contingent on data availability and coverage [[24], [25], [26]], how well variables represent reality [24], relevance and importance, reliability, validity, and set size [25,26]. Hence, to select a good representative indicator set for the regional report, criteria were developed to judge the eligibility of indicator evidence. Four criteria, two mandatory and two conditional, were used to determine indicator eligibility for the successive stage. These criteria were:

a) representativeness:
  Generally, representative *Lancet* Countdown indicators have data available for ≥80 % of countries [27]. However, SIDS have data limitations and are often underrepresented [13]. The Global Indicator Framework for SDGs uses a threshold of 50 % country inclusion to represent global progress [28]. To balance representation and data quality/availability, this threshold was adjusted to 70 % to widen the scope of inclusion.
b) suitability:
  The suitability criterion determines eligibility based on whether the variables included in an indicator fulfill their intended implication. Suitability determined the eligibility of indicator variables depending on SIDS’ context. For example, an indicator exists that monitors mortality from ambient air pollution using dose-response functions to estimate mortality from PM_2.5_ and activity from emitting sectors. However, models reflected underestimation of activity from emitting sectors in SIDS, which led to underestimation of potential mortality. Therefore, this indicator was deemed ineligible based on the suitability of one variable (sector emissions), despite the indicator being of relevance to health in SIDS (see supplementary material Table 6). Other contextual parameters, such as socio-economic or demographic differences that affect modeled data determined inclusion. It must be noted that the *Lancet* Countdown has, as a condition for indicator inclusion, measures of method accuracy, repeatability, and validity [27]. Once subject matter experts, literature review, or key knowledge determined that the variables used in indicators were suitable for the intended implication or interpretation, the eligibility condition of this sub-process was achieved.
c) temporal continuity [*conditional*]:
  In climate and clinical sciences, temporal continuity is crucial for statistical characterization, particularly for modeled data, data fluctuating over time, and other non-stationary data. Indicators with ≥80 % of years or time units (>20 % gaps in the timeline) were eligible. This applied to climate change impacts, vulnerabilities, and exposure indicators. Adaptation indicators measured whether countries have adaptive tools (e.g., adaptation plans), which would not require continuous data. Therefore, this criterion was included for indicators with continuous data for time-series analysis but not included for indicators tracking metrics cross-sectionally.
d) dataset resolution [*conditional*]:
  One major limitation of using publicly available spatial datasets is resolution. In global climate models, a parameter value can cover a 200 km x 200 km grid, which can be downscaled to 50 km^2^ or 100 km^2^. Small islands with land masses hardly exceeding 500 km^2^ may have only 2 – 4 data nodes. A limit of 0.5° x 0.5° (around 55 km^2^) was the threshold for adequate resolution. This also extends to the “resolution” of modeled health datasets without observational data from vulnerable countries, like SIDS, who may have different risk profiles than the countries normally used to establish estimates. This criterion was conditional as some indicators are from country-level self-reports (see supplementary material Table 4), rendering the limitations of “resolution” unnecessary.

Overall, the multi-criteria assessment relied on a combination of quantitative thresholds, iterative adjustment, literature review, and qualitative data retrieval techniques (Fig. 2). After this process, a set of indicators that met the criteria was extracted from the larger indicator framework.

#### 2.1.3 Cross-referencing

Both sets of indicators from stakeholder consultation and multi-criteria assessment were overlain, and overlapping indicators were obtained. This was the ideal set of indicators with relevant, reliable information on the region from which decisions can be made. However, the purpose of the method was to generate a set of indicators capable of providing a health and climate situational report for the entire region and the number of eligible indicators was too few. This was an anticipated challenge for SIDS with data limitations, and the next step was designed to adjust for this problem.

#### 2.1.4 Exemption assignment

Indicators that did not meet all criteria had to be reassessed for the exemption condition: indicator is within the prioritized areas from stakeholder consultation (described in Section 2.1.1).

This was done because more indicator evidence was needed for the purpose of reporting at a comprehensive regional level. As shown in Fig. 2, this is an iterative part of the process that backtracks to assessing ineligible indicator sets, giving precedence to those within the eight key areas at the intersection of health and climate change of relevance to SIDS [23] (see supplementary material Table 2).

Indicators meeting this criterion with < 70 % of regional coverage were included if they measured areas of relevance to the climate and health context in SIDS. However, other exempted indicators with lower than 70 % coverage required qualifiers to prevent misinterpretations of regional representativeness. For those indicators, the narrative conveyed accurate interpretation related to the suitable variable(s), stating the number of countries included and allowing for a more comprehensive report on the health and climate situation in SIDS.

### 2.2 Ranking stage

Ranking was designed to assist policymakers and/or multilateral development agencies by determining which indicators, metrics, or targets should be prioritized for action. The ranking process assigns a matrix-based relationship between two variables: data coverage and difference from global values. Data coverage refers to the proportion of countries within a region, represented as a percentage, with available indicator data. Data coverage between 70–100 % was classified as good coverage and assigned a numerical value of 5. In descending order, 50–69 % of data coverage was classified as fair, 20–49 % as low, and <20 % as very low; numerical values of 4, 3, and 2 were assigned, respectively. The second variable considered the difference between indicator-specific values from SIDS and comparable global values (see supplementary material Table 7). The degree of difference was classified into four categories, where minor differences were those with < ±5 % change and assigned a numeric of 2. In ascending order, ±5 % – ±20 % change was considered a moderate difference, ±21 % – ±40 % a high difference, and > ±40 % a very high difference. These were assigned the numeric values of 3, 4, and 5, respectively. The ranges of both variables were then categorized using a modified qualitative matrix multiplication (Eq. (1)).

*P_r_* = *C* x *Dv*

(1) *P_r_* = [*n_data_*/*n_pop_*]% x [(*V_SIDS_*-*V_global_*)/*V_global_*]%

where *P_r_* denotes prioritization rank, *C* represents data coverage, and *Dv* means the difference from global value. Matrix multiplication was chosen as it allows comparative analysis of the degree of contribution of varied factors to outcomes across multiple variables.

The equation was applied after substituting the assigned numeric values for both variables. The results corresponded to four levels of ranking: low (4–6), medium (8–10), high (12–16), and critical (20–25). In this way, the importance of selected indicators was ranked to highlight the criticality or positivity in specific capacities (Table 1). Values that showed SIDS performed better than the comparable global value, highlighted positive indicator progress. Values showing more negative implications for SIDS compared to global values highlighted areas in need of intervention or more urgent climate action.

**Table 1:** Ranking matrix method for prioritizing indicators. The product (colored cells) of numbers in brackets represent the rank level as defined in equation [1] – Prioritization Ranking (P_r_) = data coverage (C) x difference from global value (Dv) and the values in the heat map reflect this. Ranking occurred on a 4-level scale from low to critical.

## 3 Results

### 3.1 Scoping review and stakeholder consultation

#### 3.1.1 Priority areas (Relevance)

Of 47 *Lancet* Countdown indicators, 32 were within the eight priority areas from the stakeholder consultation [23] and thus were deemed eligible based on relevance to SIDS (Table 2). The majority (13 indicators) concerned impacts of extreme weather events like floods, droughts, and cyclones. Eight indicators dealt with climate-resilient health adaptation, while five tracked climate-related connections with NCDs and associated health co-benefits of climate action. Two were in prioritized areas for vector-borne diseases, investment, and scientific and political engagement.

**Table 2:** Priority Areas determined by stakeholder consultation (Allen et al. 2021) and connected Lancet Countdown indicators.

**Health and Climate Priority Areas for SIDS *(Allen* et al. *2021)***	**Eligible *Lancet* Countdown Indicators**
Impacts of Extreme Weather Events	■ exposure to warming ■ exposure of vulnerable populations to heatwaves ■ heat and physical activity ■ change in labor capacity ■ heat-related mortality ■ drought ■ extreme weather and sentiment ■ lethality of extreme weather events ■ migration, displacement, and rising sea levels ■ economic losses due to climate-related extreme events ■ value of losses due to heat-related mortality ■ loss of earnings from heat-related labor capacity reduction ■ energy systems and health
Vector-borne Diseases (VBDs)	■ climate suitability for infectious disease transmission ■ vulnerability to mosquito-borne diseases
Water, Sanitation, and Hygiene (WASH)	
Non-communicable Diseases (NCDs)	■ food insecurity ■ marine productivity ■ diet and health co-benefits ■ mortality from ambient air pollution ■ household air pollution
Collaboration on Health Adaptation	■ national assessments of climate change impacts ■ national adaptation plans for health ■ city-level climate change risk assessments ■ climate information services for health ■ air conditioning benefits and harms ■ urban green space ■ global multilateral funding for health adaptation-related funding ■ detection, preparedness, and response to health emergencies
Scientific Engagement & Investment	■ scientific engagement in health and climate change ■ corporate engagement in health and climate change
Government Engagement	■ political engagement in health and climate change ■ engagement by international organizations

### 3.2 Multi-criteria assessment

#### 3.2.1 Conditional criteria

Temporal continuity was only applied to *Lancet* Countdown Theme 1: Health Hazards, Exposures and Impacts and two connected indicators in *Lancet* Countdown Theme 4: Economics and Finance (see supplementary material Table 5); these were all eligible as >80 % of time series data were available (see supplementary material Table 4). Dataset resolution was also only applied to indicators using modeled data. Other indicators relied mostly on self-reported or observational country-level data (see supplementary material Table 4).

#### 3.2.2 Mandatory criteria

Overall, 11 indicators met the mandatory criteria of representativeness and suitability: four in Themes 1 (Health Hazards, Exposures, and Impacts) and 2 (Adaptation, Planning, and Resilience for Health), one in Theme 4 (Economics and Finance), and two in Theme 5 (Public and Political Engagement) (Table 3). This meant that indicator variables were suitable for the implications that needed to be reported on, and there were sufficient data for >70 % of countries in the SIDS region to allow for representative evidenced statements from these 11 indicators (see supplementary material Tables 3 and 6).

**Table 3:** Eligible and ineligible *Lancet* Countdown indicators based on the two mandatory criteria: representativeness and suitability.

	**Representativeness**		**Suitability**	
	(*n* = 47)		(*n* = 47)	
***Lancet* Countdown Thematic Areas**	Eligible (*n* = 11)	Ineligible (*n* = 36)	Eligible (*n* = 28)	Ineligible (*n* = 19)
**THEME 1**: Health Hazards, Exposures, and Impacts	■ exposure to warming ■ heat and physical activity ■ change in labor capacity ■ climate suitability for infectious disease transmission	■ exposure of vulnerable populations to heatwaves ■ heat-related mortality ■ wildfires ■ drought ■ extreme weather and sentiment ■ food insecurity ■ marine productivity	■ exposure to warming ■ exposure of vulnerable populations to heatwaves ■ heat and physical activity ■ change in labor capacity ■ heat-related mortality ■ climate suitability for infectious disease transmission ■ food insecurity ■ marine productivity	■ wildfires
**THEME 2:** Adaptation, Planning, and Resilience for Health	■ climate information services for health ■ global multilateral funding for health adaptation-related funding ■ lethality of extreme weather events ■ migration, displacement, and rising sea levels	■ national assessments of climate change impacts ■ national adaptation plans for health ■ city-level climate change risk assessments ■ air conditioning benefits and harms ■ urban green space ■ detection, preparedness, and response to health emergencies ■ vulnerability to mosquito-borne diseases	■ national assessments of climate change impacts ■ national adaptation plans for health ■ city-level climate change risk assessments ■ climate information services for health ■ air conditioning benefits and harms ■ urban green space ■ global multilateral funding for health adaptation-related funding ■ detection, preparedness, and response to health emergencies ■ vulnerability to mosquito-borne diseases ■ lethality of extreme weather events ■ migration, displacement, and rising sea levels	■ air conditioning benefits and harms
**THEME 3:** Mitigation Actions and Health Co-Benefits		■ energy systems and health ■ household energy use ■ sustainable and healthy road transport ■ mortality from ambient air pollution ■ household air pollution ■ emissions from agricultural production ■ diet and health co-benefits ■ healthcare sector emissions	■ energy systems and health ■ diet and co-benefits	■ household energy use ■ sustainable and healthy road transport ■ mortality from ambient air pollution ■ household air pollution ■ emissions from agricultural production ■ healthcare sector emissions
**THEME 4:** Economics and Finance	■ loss of earnings from heat-related labor capacity reduction	■ economic losses due to climate-related extreme events ■ value of losses due to heat-related mortality ■ costs of the health impacts of air pollution ■ clean energy investment ■ employment in renewable energy and fossil fuel industries ■ funds divested from fossil fuels ■ net value of fossil fuel subsidies and carbon prices ■ production- and consumption-based attribution of CO2 and PM2.5 Emissions ■ compatibility of fossil fuel company strategies with the Paris Agreement ■ fossil fuel and green bank lending	■ economic losses due to climate-related extreme events ■ value of losses due to heat-related mortality ■ loss of earnings from heat-related labor capacity reduction ■ costs of the health impacts of air pollution	■ clean energy investment ■ employment in renewable energy and fossil fuel industries ■ funds divested from fossil fuels ■ net value of fossil fuel subsidies and carbon prices ■ production- and consumption-based attribution of CO_2_ and PM_2.5_ Emissions ■ compatibility of fossil fuel company strategies with the Paris Agreement ■ fossil fuel and green bank lending
**THEME 5:** Public and Political Engagement in Health and Climate Change	■ scientific engagement in health and climate change ■ government engagement in health and climate change	■ media engagement in health and climate change ■ individual engagement in health and climate change ■ scientific engagement in health and climate change ■ engagement by international organizations ■ corporate engagement in health and climate change	■ scientific engagement in health and climate change ■ government engagement in health and climate change ■ corporate engagement in health and climate change	■ media engagement in health and climate change ■ individual engagement in health and climate change ■ engagement by international organizations

### 3.3 Cross-referencing

**Fig. 3:**
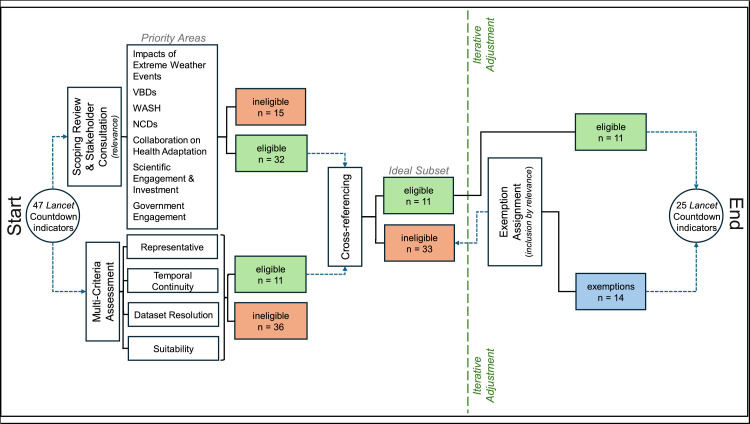
Results of the evaluation method applied to the *Lancet* Countdown Indicator Framework for the SIDS region. This graphic shows the number of indicators (n) that were progressively brought forward to obtain the 25 selected indicators for the 2024 SIDS report from the original 47 global *Lancet* Countdown indicators.

Indicators derived from the Priority Areas (Table 2, see supplementary material Table 2) were superimposed on those from the multi-criteria assessment (Table 3). All indicators from the latter were also eligible based on relevance. Therefore, these 11 indicators represented the ideal set of *Lancet* Countdown indicators that offered the most relevant metrics with enough data to make representative statements (Fig. 3).

### 3.4 Prioritization ranking

The 25 *Lancet* Countdown indicators for SIDS were then ranked based on data coverage and comparisons against global benchmarks (Table 4, see supplementary material figure 1). Aligning comparative global and SIDS values was made easier due to the similar organization of the 2023 global *Lancet* Countdown [13] and the 2024 SIDS reports [22] and availability of comparable values for 21 of the 25 selected indicators (Table 5).

Nine indicators had categorically good coverage (70–100 %), 6 had fair (50–69 %), and 6 had low data coverage (20–49 %); no selected indicators had <20 % of SIDS covered. For the variable representing the difference from global values, six indicators had positive differences (progress or less impacted) in relation to global benchmarks. One indicator had a very high positive difference (>40 %), four indicators had a high positive (±21 % to ±40 %), while one had a minor positive difference (< ±5 %).

In contrast, 14 indicators were negative as compared to global benchmarks (Table 4). The differences were very highly negative for 4, highly negative for 4, and 3 indicators each were moderately and minorly negative.

**Table 4:** Ranking of indicators that have negative implications compared to global benchmarks. n represents the number of indicators, while categories in brackets denote the level of importance. Prioritization Ranking (P_r_) = data coverage (C) x difference from global value (Dv).

To determine the need for more immediate climate action, the indicators for which SIDS were more negatively impacted or progressed less than global performance were targeted for ranking of indicator evidence (Table 4). One indicator had evidence of low, two medium, seven high, and 4 had evidence of critical importance (see supplementary material Table 7). Those 4 indicators tracked: scientific engagement in health and climate change, loss of earnings due to heat-related labor capacity reductions, food insecurity, heat and physical activity (Table 5).

**Table 5:** Comparison of indicator data at global versus SIDS levels for 21 indicators with comparable parameters. Red tones indicate likely higher impacts, worsening trends, or climate inaction. Green tones indicate likely improving trends, reduced impacts, or more climate action compared to global data. An expanded version of this table with an explanation of the parameters used for comparison for each indicator is provided in the supplementary material (pp. 10–11 - Table 7).

## 4 Discussion

In developing this method, the authors hypothesized retrieval from any global indicator framework would yield a low number of ideal indicators, which would be insufficient for technical-type reporting because low- and middle-income countries and SIDS tend to have low data coverage and quality, as well as vulnerabilities that are dissimilar to those of heavily researched countries [22,26]. Therefore, it was necessary to uncover what was *actually* usable for the region from data frameworks. However, encountering a small indicator set called for an iterative adjustment to the method. Exemptions were assigned to ineligible indicators from the multi-criteria assessment. The exemptions gave precedence to the outcome of stakeholder consultation – i.e., indicators eligible based on their intersection with the eight priority areas. The fact that there were more exemptions (*n* = 14) than eligible indicators (*n* = 11) further underscored the data limitations and research gaps pervasive across regions like SIDS. The absence resonates as a call for increasing capacities for surveillance, data capture, and integrated climate and health data frameworks.

An interesting observation highlighting the difference in lived experiences in SIDS versus global priorities was the lack of mitigation in priority areas. As the region responsible for the least greenhouse gas emissions [14], prioritizing mitigation may, understandably, seem to be of less concern to SIDS. However, the failure to include mitigation, even among subject-matter experts, also demonstrated connections between energy systems and human health, especially in the long term, have not been sufficiently evidenced. Nonetheless, most variables tracked in Theme 3 (Mitigation Actions and Health Co-Benefits) were not suitable for making implicit statements for SIDS. For example, an indicator on fossil fuel subsidies has little meaning for SIDS. Yet, sustainable development and health sector climate-resilience in SIDS is strongly determined by access to alternative sources of clean energy, and energy self-sufficiency. Indicators need to reflect this so that evidence can support investment in renewable technologies. For SIDS, this evidence may prompt adopting mitigation actions as a co-benefit rather than the target it must be for other regions. However, the bottom line remains the same: a healthy future requires the collective pursuit of green energy solutions and transitions away from fossil fuels.

One indicator (global multilateral funding for health adaptation-related funding) had a very high positive difference (> ±40 %) from comparable global values – the very high categorization of progress is understandable as these refer to Green Climate Fund financing, which is specifically dedicated to vulnerable non-SIDS and SIDS [22]. For metrics on adaptation, mitigation, or sustainable development, the isolation of “positives” is a vital component of this ranking tool, allowing policymakers to see where interventions/strategies are successful, which emboldens future climate action.

Financial and other resource constraints prevent tackling all relevant problems at once [14]. The heavy skew towards more negative indicators is expected for the region and offers a quantifiable glimpse of issues in most need of climate action. Negative implications covered a wide scope, ranging from heat, infrastructure resilience, adaptation policies, sea level rise, and engagement with climate and health issues. From indicators evidencing the most critical issues, heat arose as a recurring issue of regional importance. While there was an increase in summer temperature in 2019–2023 compared to 1985–2010 at both the global and regional levels, there was a slightly higher global increase in temperature (Table 5; indicator 1.1.1). At first glance, this may appear anomalous as heat was identified as a critical issue; however, results were likely due to the modulating effect of the ocean contributing to a smaller diurnal and annual range in temperature. This indicator is not saying that summer temperatures are higher for all regions except SIDS, but that compared to the baseline temperature, the difference is less in SIDS. In contrast, indicator 1.1.3 showed higher exposure of populations to health-threatening heat during physical activity compared to the global average (Table 5). This result was due to the year-round hours of exposure per day to higher daily temperatures for populations in SIDS.

Food insecurity tracked the influence of heatwave days and drought on the proportion of the population that reports being moderately to severely food insecure. The lack of scientific engagement, measured based on the number of peer-reviewed publications relating to climate change and health in SIDS, glaringly showed the critical need for more research on this nexus across the region.

By reviewing this evidence, policymakers and multilateral development agencies can target pertinent issues based on thematic mandates. This is not to say other issues are less actionable but offers a way to strategically allocate strained resources, addressing adaptation efforts in a staggered manner while working in conjunction with other decision-making tools. Moreover, since *Lancet* Countdown reports are annually published, there can be annual evaluation and ranking of the data in the indicator framework, which will allow policymakers to see if issues continue to be critical year-on-year. This method can aid in assessing the success/failure of any intervention at the regional level based on pre-/post-intervention assessments of criticality.

A limitation of this ranking method is that there is no distinction between a lack of available data versus minimal observable impact in countries. However, regardless of the reason for low coverage, a high proportion of countries must be included for the indicator evidence to be regionally relevant. This methodology may also lead to an underestimation of the relevance of evidence since it relies so heavily on data coverage, both in the evaluation stage and the ranking stage. Consistently low coverage should also serve as a lynchpin for action to improve data infrastructure, collection, and integration systems which are regionally lacking for SIDS and perpetuate research gaps. Furthermore, seeing the successful application of this adjunct decision-optimizing tool, there is hope that the method is adaptable to other regions with similar data and resource limitations and sociodemographic characteristics.

## 5 Conclusion

Even with evidence, taking appropriate climate action is an uphill battle for policymakers at all levels. Without evidence, appropriate action may be untenable. SIDS need assistance in being furnished with the tools that provide irrefutable evidence across all areas. Emphasis must also be placed on securing inter-regional data-sharing networks so that scientific research can be undertaken without onerous bureaucratic barriers.

## CRediT authorship contribution statement

**Stephanie Y Parker:** Writing – review & editing, Writing – original draft, Visualization, Methodology, Formal analysis. **Kimalie F Parchment:** Writing – review & editing, Methodology, Formal analysis. **Maria Walawender:** Writing – review & editing, Validation, Methodology, Data curation. **Georgiana Gordon-Strachan:** Writing – review & editing, Supervision, Project administration, Methodology, Conceptualization.

## Declaration of competing interest

The authors declare that they have no known competing financial interests or personal relationships that could have appeared to influence the work reported in this paper.

## Appendix Supplementary materials

Image, application 1

## References

[1] Gulev S.K., Thorne P.W., Ahn J., Dentener F.J., Domingues C.M., Gerland S., Osborn T.J., Zarrin A. (2021). Climate change 2021 – the physical science basis: working group i contribution to the sixth assessment report of the intergovernmental panel on climate change.

[2] Food and Agriculture Organization (2018). https://openknowledge.fao.org/server/api/core/bitstreams/9aeb8ade-a623-4954-8adf-204daae3b5de/content.

[3] Mora C., McKenzie T., Gaw I.M., Dean J.M., von Hammerstein H., Knudson T.A. (2022). Over half of known human pathogenic diseases can be aggravated by climate change. Nat Clim Chang.

[4] Health Policy Watch (2023). Small island developing states at nexus of climate, unhealthy foods and mental health challenges. https://healthpolicy-watch.news/small-island-developing-states-at-nexus-of-climate-unhealthy-foods-and-mental-health-challenges/.

[5] Lee H., Calvin K., Dasgupta D., Krinner G., Mukherji A., Thorne P., Lee H., Romero J. (2023). Climate change 2023: synthesis report. contribution of working groups I, ii and iii to the sixth assessment report of the intergovernmental panel on climate change.

[6] Kelman I., Ayeb-Karlsson S., Rose-Clarke K., Prost A., Ronneberg E., Wheeler N. (2021). A review of mental health and wellbeing under climate change in small island developing states (SIDS). Environ Res Lett.

[7] Watts N., Adger W.N., Agnolucci P., Blackstock J., Byass P., Cai W. (2015). Health and climate change: policy responses to protect public health. Lancet.

[8] Conference of the Parties to the United Nations Framework Convention on Climate Change (21st session: 2015: Paris) (2015). https://www.un.org/en/development/desa/population/migration/generalassembly/docs/globalcompact/FCCC_CP_2015_10_Add.1.pdf.

[9] World Health Organization (2021). https://iris.who.int/bitstream/handle/10665/348068/9789240038509-eng.pdf.

[10] Thomas A., Baptiste A., Martyr-Koller R., Pringle P., Rhiney K. (2020). Climate change and small island developing states. Annu Rev Env Resour.

[11] Sachs J., Massa I., Marinescu S., Lafortune G. (2022). https://files.unsdsn.org/WP_MVI_Sachs%20Massa%20Marinescu%20Lafortune_FINAL_cVeeBVmKSKyYYS6OyiiH.pdf.

[12] Mycoo M., Wairiu M., Campbell D., Duvat V., Golbuu Y., Maharaj S., Agard J., Riyaz M. (2022). Climate change 2022: impacts, adaptation and vulnerability. contribution of working group ii to the sixth assessment report of the intergovernmental panel on climate change.

[13] Romanello M., di Napoli C., Green C., Kennard H., Lampard P., Scamman D. (2023). The 2023 report of the Lancet countdown on health and climate change: the imperative for a health-centred response in a world facing irreversible harms. Lancet.

[14] United Nations Trade and Development (2022). https://unctad.org/system/files/official-document/ciiem6d2_en.pdf.

[15] Costello A., Abbas M., Allen A., Ball S., Bell S., Bellamy R. (2009). Managing the health effects of climate change: lancet and university college London institute for global health commission. Lancet.

[16] Mays N., Pope C., Popay J. (2005). Systematically reviewing qualitative and quantitative evidence to inform management and policy-making in the health field. J Health v Res Policy.

[17] Hanney S.R., Gonzalez-Block M.A., Buxton M.J., Kogan M. (2003). The utilisation of health research in policy-making: concepts, examples and methods of assessment. Health Res Policy Syst.

[18] Rahman M.S. (2016). The advantages and disadvantages of using qualitative and quantitative approaches and methods in language “testing and assessment” research: a literature review. J Educ Learn.

[19] Hamilton A.B., Finley E.P. (2019). Qualitative methods in implementation research: an introduction. Psychiatry Res.

[20] Sofaer S. (1999). Qualitative methods: what are they and why use them?. Health v Res.

[21] Anderson C. (2010). Presenting and evaluating qualitative research. Am J Pharm Educ.

[22] Gordon-Strachan G.M., Parker S.Y., Harewood H.C., Méndez-Lázaro P.A., Saketa S.T., Parchment K.F. (2025). The 2024 small island developing states report of the Lancet Countdown on health and climate change. Lancet Glob Health.

[23] Allen C., West R., Beagley J., McGushin A. (2021). https://lancetcountdown.org/wp-content/uploads/2024/03/Climate-Change-and-Health-in-Small-Island-Developing-States_February-2022.pdf.

[24] Munda G., Nardo M. (2003). https://www.researchgate.net/publication/268048297_On_the_Methodological_Foundations_of_Composite_Indicators_Used_for_Ranking_Countries.

[25] Schang L., Blotenberg I., Boywitt D. (2021). What makes a good quality indicator set? A systematic review of criteria. Int J Qual Health Care.

[26] Veillard J., Cowling K., Bitton A., Ratcliffe H., Kimball M., Barkley S. (2017). Better measurement for performance improvement in low-and Middle-income countries: the primary health care performance Initiative (PHCPI) experience of conceptual framework development and indicator selection. Milbank Q.

[27] Di Napoli C., McGushin A., Romanello M., Ayeb-Karlsson S., Cai W., Chambers J. (2022). https://bmcpublichealth.biomedcentral.com/articles/10.1186/s12889-022-13055-6.

[28] United Nations (2019). Tier classification for global SDG indicators. https://mdgs.un.org/sdgs/files/Tier_Classification_of_SDG_Indicators_20_November_2019_web.pdf.

