# Supplementary material for: Prioritizing evidence for action from the 2024 small island developing states report of the Lancet Countdown on health and climate change
Source: J Clim Chang Health. 2025 Jul 23;24:100482. doi: 10.1016/j.joclim.2025.100482 (PMC12851233; doi:10.1016/j.joclim.2025.100482)
Supplement: Supplementary file 1 [file mmc1.docx]

# Supplementary Material for “Prioritizing evidence for action from the 2024 Small Island Developing States report of the Lancet Countdown on Health and Climate Change”

The Journal of Climate Change and Health

Manuscript Number: **JOCLIM-D-24-00187**  
Prioritizing evidence for action from the 2024 Small Island Developing States report of the Lancet Countdown on Health and Climate Change

**Table 1** List of the 59 small island developing states by sub-region

| **Atlantic and Indian Oceans and South China Seas** | **Caribbean** | **Pacific** |
| --- | --- | --- |
| Cabo Verde, Comoros, Guinea-Bissau, Maldives, Mauritius, São Tomé and Príncipe, Seychelles, Singapore | Anguilla, Antigua and Barbuda, Aruba, Bahamas, Barbados, Belize, Bermuda, Bonaire, British Virgin Islands, Cayman Islands, Cuba, Curacao, Dominica, Dominican Republic, Grenada, Guadeloupe, Guyana, Haiti, Jamaica, Martinique, Montserrat, Puerto Rico, Saint Kitts and Nevis, Saint Lucia, Saint Martin, Saint Vincent and the Grenadines, Sint Maarten, Suriname, Trinidad and Tobago, Turks and Caicos Islands, U.S. Virgin Islands | American Samoa, Commonwealth of Northern Marianas, Cook Islands, Federated States of Micronesia, Fiji, French Polynesia, Guam, Kiribati, Marshall Islands, Nauru, New Caledonia, Niue, Palau, Papua New Guinea, Samoa, Solomon Islands, Timor-Leste, Tonga, Tuvalu, Vanuatu |

**Table 2** Eight Priority Indicator Areas derived from the stakeholder consultation as described in the Method Section.

(Source: Allen, C., R. West, J. Beagley and A. McGushin (2021). **Climate Change and Health in Small Island Developing States, The Lancet Countdown on Health and Climate Change**: 1-65.)

| **Priority Areas for SIDS** | | **Relevance** |
| --- | --- | --- |
| **1** | **Health impacts of severe weather events** | Health impacts following cyclones include injuries and illness from communicable diseases and NCDs resulting from damage to infrastructure and health systems. Monitoring of other events such as heatwaves, floods and drought is needed. |
| **2** | **Vector-borne diseases (VBDs)** | Weak sanitation systems, warmer temperatures and severe and unpredictable weather patterns in SIDS promote VBD spread. SIDS in the Caribbean and Pacific are now affected by four dengue variants, often simultaneously. |
| **3** | **Water, sanitation and hygiene (WASH)** | Access to clean water is threatened by saltwater intrusion of aquifers, damage to infrastructure from severe weather events, and contamination as pathogens proliferate. |
| **4** | **Non-communicable diseases (NCDs)** | Losses in biodiversity and local food production result in increased reliance on processed foods. Environmental determinants affect cardiovascular and respiratory health. |
| **5** | **Collaboration between agencies** | Adaptation must involve cooperation between the health sector and other sectors, especially agencies responsible for planning and infrastructure, disaster management and meteorological forecasting. |
| **6** | **Research and surveillance linking climate and health** | Research and surveillance are challenged by factors, such as small population sizes and limited institutional cooperation. Strong health surveillance is needed for modelling. |
| **7** | **Investment in surveillance and research** | There is a lack of dedicated funding for routine data capture, analysis and reporting in SIDS. Academic programmes are needed to build human resources. |
| **8** | **Government engagement in health and climate change** | SIDS governments have achieved prominence on the world stage in the discussion of impacts of climate change. There is a need for measurement of action to adapt systems to prevent and treat ill-health arising from climate change. |

**Table 3** Lancet Countdown indicator coverage. **Representativeness** for SIDS as a region is met when indicator data are available for at least 70% of countries. Not all 47 indicators from the Lancet Countdown Indicator Framework are shown below.

| ***Lancet* Countdown Indicators** | | Pacific  (n =20) | Caribbean (n=31) | AIS  (n=9) | Coverage |
| --- | --- | --- | --- | --- | --- |
| ***Health Hazards, Exposures, and Impacts*** | | | | | |
| 1.1.1 | exposure to warming | 19(95%) | 30(97%) | 8(89%) | **94%** |
| 1.1.2 | exposure of vulnerable populations to heatwaves | 9(45%) | 14(45%) | 8(89%) | **60%** |
| 1.1.3 | heat and physical activity | 20(100%) | 28(90%) | 8(89%) | **93%** |
| 1.1.4 | change in labour capacity | 20(100%) | 28(90%) | 8(89%) | **93%** |
| 1.1.5 | heat-related mortality | 10(50%) | 13(42%) | 5(56%) | **49%** |
| 1.2 | drought | 6(30%) | 6(19%) | 2(22%) | **24%** |
| 1.3 | climate suitability for infectious disease transmission | 7(35%) | 22(71%) | 6(67%) | **58%** |
| 1.4.1 | food security and undernutrition |  |  |  |  |
| 1.4.2 | marine food insecurity |  |  |  |  |
| ***Adaptation, Planning, and Resilience for Health*** | | | | | |
| 2.1 | assessment and planning of health adaptation | 20% | 29% | 22% | **24%** |
| 2.2.1 | climate information services for health | 75% | 61% | 78% | **71%** |
| 2.2.2 | urban green space | 20% | 35% | 67% | **41%** |
| 2.2.3 | health adaptation-related funding | 75% | 52% | 100% | **76%** |
| 2.2.4 | detection, preparedness, and response to health emergencies | 40% | 52% | 100% | **64%** |
| 2.3.1 | lethality of extreme weather events | 95% | 87% | 67% | **83%** |
| 2.3.2 | migration, displacement and rising sea levels | 40% | 10% | 22% | **24%** |
| ***Mitigation Actions and Co-Benefits*** | | | | | |
| 3.1.1 | carbon intensity of the energy system |  |  |  |  |
| 3.1.2 | coal phase out | not available for SIDS | | | |
| 3.3.1 | agricultural emissions | 45% | 55% | 89% | 63% |
| 3.5.2 | diet and health co-benefits | 40% | 48% | 89% | 59% |
| ***Economics and Finance*** | | | | | |
| 4.1.1 | economic losses due to climate-related extreme events |  |  |  |  |
| 4.1.2 | costs of heat-related mortality | 6(30%) | 12(39%) | 5(56%) | **41%** |
| 4.1.3 | loss of earnings from heat-related labour capacity loss | 13(65%) | 20(65%) | 9(100%) | **77%** |
| 4.2.1 | clean energy investment | unavailable for SIDS | | | |
| 4.2.4 | net value of fossil fuel subsidies and carbon prices | 0% | 1(3%) | 1(11%) | **5%** |
| ***Public and Political Engagement*** | | | | | |
| 5.3 | scientific engagement in health and climate change | 19 (95%) | 22 (71%) | 9 (100%) | **89%** |
| 5.4.1 | government engagement in health and climate change (UNGA) | 13 (65%) | 16 (52%) | 7 (78%) | **65%** |
| 5.4.2 | government engagement in health and climate change (NDCs) | 15 (75%) | 16 (52%) | 8 (89%) | **72%** |
| 5.5 | corporate engagement in health and climate change | 1 (5%) | 4 (13%) | 7 (78%) | **32%** |

**Table 4** List of datasets used by Lancet Countdown Indicators, their resolution, and eligibility

| **Datasets** | **Resolution** | **Eligibility** |
| --- | --- | --- |
| ***Health Hazards, Exposures, and Impacts*** | | |
| ERA5 reanalysis | 28km^2^ | Adequate |
| ISIMIP Gridded Population | 55km^2^ | Adequate |
| Hybrid gridded demographic data | 28km^2^ | Adequate |
| SEDAC Gridded Population |  | Adequate |
| ILOSTAT data |  | Adequate |
| SPEI | 110km^2^ | Inadequate |
| ERA5-Land reanalysis | 55km^2^ | Adequate |
| HYDE 3.2 gridded population |  | Adequate |
| JISAO Altitude Data | 55km | Adequate |
| Copernicus Land Cover | 55km2 | Adequate |
| ***Adaptation, Planning, and Resilience for Health*** | | |
| Landstat Satellite Data from NASA-USGS Programme | 1km^2^ | Adequate |
| Center for International Earth Science Information Network (Columbia University) | 30-arcsecond output | Adequate |
| Global Human Settlement Programme of the European Commission (GHS) |  | Adequate |
| EM-DAT at the Centre for Research on the Epidemiology of Disasters (CRED) at the Université Catholique de Louvain, | Country Level | Adequate |
| Human Development Index (HDI) at the United Nations Development Programme, Human Development Report | Country Level | Adequate |
| IHR | Country Level | Adequate |
| Coastal Digital Elevation Model | Global (90m) | Adequate |
| Estimated global mean increases in sea | Global | Adequate |
| Hybrid gridded demographic data for the world |  | Adequate |
| ***Mitigation Actions and Co-Benefits*** | | |
| FAOSTAT database | Country Level | Adequate |
| Global Dietary Database (dietary intake)** | 114-country survey | Inadequate |
| Adult Body-mass Index in 200 Countries from 1975 to 2014** | Four Continents | Inadequate |
| ***Economics and Finance*** | | |
| EM-DAT The International Disaster Database (Centre for Research on the Epidemiology of Disasters) | Country Level | Adequate |
| IMF World Economic Outlook | Country Level | Adequate |
| Fossil fuel subsidies data (IEA) | Country Level | Adequate |
| Carbon pricing data (World Bank Carbon Pricing Dashboard) | Country Level | Adequate |
| Health expenditure data (WHO) | Country Level | Adequate |
| Lancet Countdown Indicator 1.1.5 | Country Level | Adequate |
| Lancet Countdown Indicator 1.1.4 | Country Level | Adequate |
| Energy sources for Electricity Generation (IRENA) | Country Level | Adequate |
| US Dollar GDP deflator index (IMF) | Country Level | Adequate |
| VSL in OECD are taken from OECD report on Mortality Risk Valuation in Environment, Health and Transport Policies. | Country Level | Adequate |
| Years of remaining life (WHO) | Global Survey | Adequate |
| World average GDP per capita in current USD and inflation rate each year | Global | Adequate |
| Data on earnings by country and sector from ILOSTAT | Country Level | Adequate |
| Exchange rate data (IMF) | Country Level | Adequate |
| US Dollar CPI and GDP deflator index (IMF) | Country Level | Adequate |
| Country GDP data from the IMF World Economic Outlook database (IMF) | Country Level | Adequate |
| World Bank Income Groups | Country Level | Adequate |
| ***Public and Political Engagement*** | | |
| Bibliographic Index Database (OpenAlex) | Country Keyword | Adequate |
| Nationally Determined Contributions Reports (UNFCCC) | Country Level | Adequate |
| Country Statements (UN General Database Corpus) | Country Level | Adequate |
| Global Compact Communication of Progress Reports (UN) | Individual Companies | Adequate |

**References: Global Food Losses and Food Waste ***Gustavsson,*** ***J et al***

Consumption of Nuts & Legumes & Risk of Incident Ischemic Heart Disease, Stroke, & Diabetes, ***Afshin, A et al.***

Food Groups and Risk of Coronary Heart Disease, Stroke and Heart Failure ***Bechthold***, ***A et al***

Food Groups and Risk of Colorectal Cancer ***Schwingshackl, L et al***.

Food Groups and Risk of Type 2 Diabetes Mellitus ***Schwingshackl, L et al***

Risk-disease associations: Bradford-Hill criteria (NutriCoDE), ***World-Cancer-Research-Fund criteria (GBD), & NutriGrade***.

**Table 5** Temporal continuity of Lancet Countdown Indicators.

| *Lancet* Countdown Indicators | | Time Frame | Duration (years) | Max. Continuous Years* |
| --- | --- | --- | --- | --- |
| ***Health Hazards, Exposures, and Impacts*** | | | | |
| 1.1.1 | exposure to warming | 2000-2022 | 23 | 23 |
| 1.1.2 | exposure of vulnerable populations to heatwaves | 1980-2022 | 43 | 23(19) |
| 1.1.3 | heat and physical activity | 2000-2022 | 23 | 23 |
| 1.1.4 | change in labour capacity | 1990-2022 | 33 | 33 |
| 1.1.5 | heat-related mortality | 2000-2022 | 23 | 23 |
| 1.2.2 | drought | 1950-2022 | 73 | Intermittent |
| 1.3 | climate suitability for infectious disease transmission | 1951-2022 | 72 | 72 |
| 1.4.2 | food security and undernutrition | 1982-2022 | 41 | 41 |
| ***Adaptation, Planning, and Resilience for Health*** | | | | |
| 2.1 | assessment and planning of health adaptation | 2021 |  |  |
| 2.2.1 | climate information services for health | 2021 |  |  |
| 2.2.3 | urban green space | 2015-2022 | 8 | 2020-2022 |
| 2.2.4 | health adaptation-related funding | 2015-2023 | 9 | 2015-2022 |
| 2.2.5 | detection, preparedness, and response to health emergencies | 2023 | 1 |  |
| 2.3.2 | lethality of extreme weather events | 1900-2023 | 124 | selected 2000-2022 |
| 2.3.3 | migration, displacement and rising sea levels | 2022 | 1 |  |
| ***Mitigation Actions and Co-Benefits*** | | | | |
| 3.1.1 | carbon intensity of the energy system | 2000-2023 |  |  |
| 3.1.2 | coal phase out |  |  |  |
| 3.3.1 | agricultural emissions | 1995-2020 | 26 | 1995-2020* |
| 3.5.2 | diet and health co-benefits | 2019-2020 | 2 | 2019-2020 |
| ***Economics and Finance*** | | | | |
| 4.1.1 | economic losses due to climate-related extreme events | 2010-2022 | 13 | 2021-2022 |
| 4.1.2 | costs of heat-related mortality | 2000-2022 | 23 | 2000-2022 |
| 4.1.3 | loss of earnings from heat-related labour capacity Loss | 1990-2022 | 33 | 1990-2022 |
| 4.2.1 | clean energy investment |  |  |  |
| 4.2.4 | net value of fossil fuel subsidies and carbon prices | 2010-2022 | 12 | 2010-2022 |
| 4.2.5 | production- and consumption-based attribution of CO_2_ and PM2.5 Emissions |  |  |  |
| ***Public and Political Engagement*** | | | | |
| 5.3 | scientific engagement in health and climate change | 1990-2022 | n/a | 33 |
| 5.4.1 | government engagement in health and climate change (UNGA) | 2022 | n/a | 1 |
| 5.4.2 | government engagement in health and climate change (NDCs) | First & second submissions | | |
| 5.5 | corporate engagement in health and climate change | 2011-2023 | 12 | 12 |

*Maximum years for which data are available for all countries

**Table 6** Suitability of Lancet Countdown Indicators to 2024 SIDS Report – relevance of indicators to discourse.

| ***Lancet* Countdown Indicators** | | **Data Type** | **Climate Parameter(s)** | **Impact Variable(s)** | **Impact Expression** | **Intended Analysis** | **Discourse Implication(s)** |
| --- | --- | --- | --- | --- | --- | --- | --- |
| ***Health Hazards, Exposures, and Impacts*** | | | | | | | |
| 1.1.1 | exposure to warming | Modelled | Temperature | Location | local change from baseline summer temperature | Δhazard coverage | impact on vulnerability of localities |
| 1.1.2 | exposure of vulnerable populations to heatwaves | Inferred | Temperature | Infants under 1  People over 65 | person hours of exposure | Δexposure & vulnerability | impact on activity, health, and HC access of vulnerable |
| 1.1.3 | heat and physical activity | Inferred | Temperature; Humidity | Persons engaged in light or moderate activity | Heat stress risk | Δrisk of heat stress | impact on outdoor activities, occupational hazards |
| 1.1.4 | change in labour capacity | Inferred | Temperature; Humidity | Labour force in agriculture, industry, service, construction | Work hours lost | Δrisk of heat stress | impact on social and health inequity, & productivity |
| 1.1.5 | heat-related mortality | Inferred | Temperature | > 65 population | heat-attributed excess deaths | Δrisk of mortality | impact of excess deaths from temperature |
| 1.2.2 | drought | Modelled | Precipitation; Evapotranspiration | Land cover | Drought-Affected Area | Δhazard coverage & intensity | impact on nutritional patterns, sanitation, water security |
| 1.3 | climate suitability for infectious disease transmission | Modelled | Precipitation; Temperature | Dengue  Malaria  *Vibrio cholerae*  West Nile Virus | R0 LTS  Vibrio (km^2^)  R0 | Δhazard coverage | impact of infectious disease transmission |
| 1.4.2 | food security and undernutrition | Inferred | Temperature  SPEI | Food insecurity experience scale (FIES) | Moderate to severe food insecurity | Δfood insecure population | impact of drought & heatwaves on reports of being food insecure |
| ***Adaptation, Planning, and Resilience for Health*** | | | | | | | |
| 2.1 | assessment and planning of health adaptation | Self-reported | General Hydromet Hazards | NAP w/ CC&H  w/ CC&H funds  CC&H VRA | Yes or No | w/ NAP-based resource allocation | impact on mitigation actions |
| 2.2.1 | climate information services for health | Self-reported | CSDs | Surveillance of CSDs  Met-integrated CSD Surveillance  Early Warning Systems | Yes or No | State of information systems | impact on preparedness and mitigation |
| 2.2.3 | urban green space | Modelled |  | Peak NDVI  Population-weighted peak NDVI | Green Space | Δresilience | impact on resilience to thermal radiation |
| 2.2.4 | health adaptation-related funding | Actual | General Hydromet Hazards | Readiness Activities Financing  Funding Activities | Allocated USD | availability of external funding | Progress of commitments to assist SIDS |
| 2.2.5 | detection, preparedness, and response to health emergencies | Self-reported | General | Planning for Health Emergencies  Manag. of Health Emergencies  Emergency Logistics & Supply | IHR Scores (0-100%) | Potential capacity of HC systems | overall resilience, level of CCAP for Health |
| 2.3.2 | lethality of extreme weather events | Actual | Flooding  Drought  Landslides  Hurricanes | Deaths  Affected  Damage | Total Persons & Cost | Δ adverse outcomes | Impact on wellbeing and life expectancy |
| 2.3.3 | migration, displacement and rising sea levels | Modelled | Sea level rise | Coastal areas | Population in 1m of coast | Δhazard exposure | Implication for displacement; planning for SLR displacement |
| ***Mitigation Actions and Co-Benefits*** | | | | | | | |
| 3.1.1 | carbon intensity of the energy system | Indicator data unavailable for SIDS. New indicator developed for SIDS | | | | | |
| 3.1.2 | coal phase out | Indicator data unavailable for SIDS | | | | | |
| 3.3.1 | agricultural emissions | Modelled | Livestock  Agricultural Products  Dairy | N_2_O emission | MtCO_2_e Production | ΔGHG emissions from consumption | impact of consumption and food demand on GHGs and associated outcomes |
| 3.5.2 | diet and health co-benefits | Inferred | Baseline food consumption  Baseline Weight | Relative Risk  GBD Mortality | Diet-related deaths | Δadverse outcomes | Impact of nutritional patterns on mortality |
| ***Economics and Finance*** | | | | | | | |
| 4.1.1 | economic losses due to climate-related extreme events | Actual & Estimated | Weather-related Disasters | Cost | Uninsured Loss  Insured Loss  Proportion of GDP | Δexpense of CC | impact on financial capacity, and budgetary planning |
| 4.1.2 | costs of heat-related mortality | Inferred | Heat-related mortality (Indicator 1.1.5) | Value of statistical life-year (over 65 yo) | Monetary value of YLL | Δlosses from temperature | expense of non-corrective measures; |
| 4.1.3 | loss of earnings from heat-related labour capacity Loss | Inferred | Temperature; Humidity (work hours lost – 1.1.4) | Agriculture, manufacturing, and construction sectors | Cost of work hours lost | Δlosses from temperature | expense of non-corrective measures; |
| 4.2.1 | clean energy investment | Indicator data unavailable for SIDS | | | | | |
| 4.2.4 | net value of fossil fuel subsidies and carbon prices | Actual | Net Carbon Revenue  Net Carbon Price  Net Carbon Revenue/Health Expenditure (%) | Δinvestment in fossil fuels | impact on transitioning to net zero, implication for subsidy reform |  |  |
| 4.2.5 | production- and consumption-based attribution of CO_2_ and PM2.5 Emissions | Dataset (based on indicator 3.3.1) deemed ineligible | | | | | |
| ***Public and Political Engagement*** | | | | | | | |
| 5.3 | scientific engagement in health and climate change | Published | General Hydrometeorologic Hazards | Adaptation  Impacts  Mitigation | Count over time |  | changing knowledge base for SIDS; implication on capacity for research |
| 5.4.1 | government engagement in health and climate change (UNGA) | Submitted | General Hydrometeorologic Hazards | Climate  Health  Climate and Health | Count over time |  | calls for support; persistence of issues |
| 5.4.2 | government engagement in health and climate change (NDCs) | Submitted | General Hydrometeorologic Hazards | Climate-sensitive illnesses and outcomes | Inclusion  (Yes or No) |  | intention to prioritize; prevalence and persistence of issues |
| 5.5 | corporate engagement in health and climate change | Submitted | General Hydrometeorologic Hazards | Climate  Health  Climate and Health | Count and Proportion |  | visibility of issues to corporations; intention to use sustainable practices |

**Table 7** Comparison of indicator data at global versus SIDS levels for 21 indicators with comparable parameters. Red tones may indicate higher impacts, worsening trends, or climate inaction. Green tones may indicate improving trends, reduced impacts, or more climate action compared to global data.

| *Lancet* Countdown Indicators used in the 2024 SIDS Report of the *Lancet* Countdown on Health and Climate Change | Data Availability | Coverage Bracket | Global Value | SIDS Value | Global - SIDS (Percentage Change) | Prioritization Ranking | Importance |
| --- | --- | --- | --- | --- | --- | --- | --- |
| ***Health Hazards, Exposures, and Impacts*** | | | | | | | |
| **1.1.1 Exposure to warming** change in summer temperature in 2022 from 1986-2005 baseline (^o^C) | 97% | Good | 0.3 | 0.2 | -33% | High (positive) |  |
| **1.1.2 exposure of vulnerable populations to heatwaves** percentage change in average heatwave days per vulnerable person (2013-2022 vs 1986-2005) | 63% | Fair | 9.1 | 7.89 | -13% | High (positive) |  |
| **1.1.3 heat and physical activity** change in annual hours with potential for at least moderate heat stress risk during light intensity physical activity in 1991-2000 vs 2013-2022 | 97% | Good | 241 | 336 | 39% | High (negative) | Critical |
| **1.1.4 change in labour capacity** percentage change in loss of potential labour hours in 2022 vs 1991-2000 | 97% | Good | 42 | 40 | -5% | Minor (negative) |  |
| **1.1.5 heat-related mortality** days of health-threatening high temperatures annually in 2018-2022 | 51% | Fair | 86 | 103 | 20% | Moderate (negative) | High |
| **1.2 drought** coverage of land area experiencing at least 1-month extreme drought | 25% | Low | 47 | 73 | 55% | Very High (negative) | High |
| **1.3 climate suitability for iinfectious disease transmission: dengue** percentage change of R0 in 1951-60 vs 2013-22 | 95% | Good | 28.2 | 33 | 17% | Moderate (negative) | High |
| **1.4.1 food insecurity** percentage of additional people experiencing food insecurity due to heatwave days and drought in 2021 vs 1981-2010 | 56% | Fair | 1.6 | 4.2 | 163% | Very High (negative) | Critical |
| **1.4.2 marine productivity** change in 3-yr mean sea surface temperature in 2021 vs 1981-1990 | 47% | Low | 0.476 | 0.484 | 2% | Minor (negative) | Low |
| ***Adaptation, Planning, and Resilience for Health*** | | | | | | | |
| **2.1.1 national assessments of climate change impacts** percentage of countries with national climate assessments by 2021 | 41% | Low | 17 | 4 | -76% | Very High (negative) | High |
| **2.1.2 national adaptation plans for health** percentage of countries with national adaption plans | 41% | Low | 6 | 8 | 33% | High (positive) |  |
| **2.2.1 climate information services for health** proportion of countries with meteorological data services for health | 77% | Good | 50 | 40 | -20% | Moderate (negative) | High |
| **2.2.3 health adaptation-related funding** proportion of funds given to projects with potential health benefits | 76% | Good | 17.7 | 50 | 182% | Very High (positive) |  |
| **2.2.4 detection, preparedness, and response to health emergencies** percentage of countries with high to very high IHR 7 capacities | 64% | Fair | 70 | 72 | 3% | Minor (positive) |  |
| ***Mitigation Actions and Co-Benefits*** | | | | | | | |
| **3.3.2 diet and health co-benefits** percentage change in deaths attributable to high-emission-worsening dietary risks in 2022 vs 2019 | 63% | Fair | 2 | 2 | Same as global | | |
| ***Economics and Finance*** | | | | | | | |
| **4.1.2 value of losses due heat-related mortality** percentage change of 2000-2004 vs 2018-2022 averages of average annual income losses | 44% | Low | 149 | 190 | 28% | High (negative) | High |
| **4.1.3 loss of earnings from heat-related labour capacity reduction** potential loss in earnings as a percentage of avg GDP in 2022 | 77% | Good | 0.87 | 2.66 | 206% | High (negative) | Critical |
| ***Public and Political Engagement*** | | | | | | | |
| **5.1 scientific engagement in health and climate change** ratio of articles to counties (pooled) in 2022 | 89% | Good | 16.1 | 0.9 | -94% | Very High (negative) | Critical |
| **5.2 government engagement in health and climate change (UNGA)** proportion of countries that mentioned health-climate intersection in 2022 UNGA Debate | 68% | Fair | 50 | 35 | -30% | High (negative) | High |
| **5.2 government engagement in health and climate change (NDCs)** percentage of countries including health in updated NDCs | 76% | Good | 95 | 94 | -1% | Minor (negative) | Medium |
| **5.3 corporate engagement in health and climate change** proportion of companies that mentioned health and climate in 2022 | 35% | Low | 38 | 52 | 37% | High (positive) |  |

**Theme 1: Health hazards, exposures, and impacts**

1**.**1 Health and heat

1.1.1 Exposure to heating

1.1.2 Exposure of vulnerable populations to heatwaves

1.1.3 Heat and physical activity

1.1.4 Change in labour capacity

1.1.5 Heat-related mortality

1.2 Health and extreme weather events

1.2.1 Drought

1.3 Climate suitability for infectious disease transmission

1.4 Food security and undernutrition

1.4.1 Food Insecurity

1.4.2 Marine Food Insecurity

**Theme 2: Adaptation, planning, and resilience for health**

2.1 Assessment and planning of health adaptation

2.1.1 National assessments of climate change impacts, vulnerability, and adaptation for health

2.1.2 National adaptation plans for health

2.2 Enabling conditions, adaptation delivery, and implementation

2.2.1 Climate information for health

2.2.2 Urban greenspace

2.2.3 Health adaptation-related funding

2.2.4 Detection, preparedness, and response to health emergencies

2.3 Vulnerabilities, health risk, and resilience to climate change

2.3.1 Lethality of extreme weather events

2.3.2 Migration, displacement, and rising sea levels

**Theme 3: Mitigation actions and health co-benefits**

3.1 Energy use, energy generation and health

3.2 Diet and health co-benefits

**Theme 4: Economics and finance**

4.1 Economic impact of climate change and its mitigation

4.1.1 Economic losses due to climate-related extreme events

4.1.2 Costs of heat-related mortality

4.1.3 Loss of earnings from heat-related labour capacity loss

**Theme 5: Public and political engagement**

5.1 Scientific engagement in health and climate change

5.2 Government engagement in health and climate change

5.3 Corporate sector engagement in health and climate change

**Figure 1** The indicators of the 2024 SIDS report of the Lancet Countdown. (Source: Gordon-Strachan GM, Parker SY, Harewood HC, Méndez-Lázaro PA, Saketa ST, Parchment KF, Walawender M, Abdulkadri AO, Beggs PJ, Buss DF, Chodak RJ. The 2024 small island developing states report of the Lancet Countdown on health and climate change. The Lancet Global Health. 2025 Jan 1;13(1):e146-66. https://doi.org/10.1016/S2214-109X(24)00421-2)
